# Use of community-level data in the National Children’s Study to establish the representativeness of segment selection in the Queens Vanguard Site

**DOI:** 10.1186/1476-072X-11-18

**Published:** 2012-06-05

**Authors:** Andrew Rundle, Virginia A. Rauh, James Quinn, Gina Lovasi, Leonardo Trasande, Ezra Susser, Howard F. Andrews

**Affiliations:** 1 Department of Epidemiology, Mailman School of Public Health, 722 West 168th Street, New York, NY, 10032, USA; 2 Heilbrunn Center for Population & Family Health, 60 Haven Ave B-3, New York, NY, 10032, USA; 3 Institute for Social and Economic Research and Policy, 420 West 118th Street, New York, NY, 10027, USA; 4 Departments of Pediatrics and Environmental Medicine, NYU School of Medicine and NYU Wagner School of Public Service,; 5 Department of Biostatistics, Mailman School of Public Health, 722 West 168th Street, New York, NY, 10032, USA

**Keywords:** Neighborhood health, Social environment, Built environment, Children, Study design

## Abstract

**Background:**

The WHO Multiple Exposures Multiple Effects (MEME) framework identifies community contextual variables as central to the study of childhood health. Here we identify multiple domains of neighborhood context, and key variables describing the dimensions of these domains, for use in the National Children’s Study (NCS) site in Queens. We test whether the neighborhoods selected for NCS recruitment, are representative of the whole of Queens County, and whether there is sufficient variability across neighborhoods for meaningful studies of contextual variables.

**Methods:**

Nine domains (demographic, socioeconomic, households, birth rated, transit, playground/greenspace, safety and social disorder, land use, and pollution sources) and 53 indicator measures of the domains were identified. Geographic information systems were used to create community-level indicators for US Census tracts containing the 18 study neighborhoods in Queens selected for recruitment, using US Census, New York City Vital Statistics, and other sources of community-level information. Mean and inter-quartile range values for each indicator were compared for Tracts in recruitment and non-recruitment neighborhoods in Queens.

**Results:**

Across the nine domains, except in a very few instances, the NCS segment-containing tracts (N = 43) were not statistically different from those 597 populated tracts in Queens not containing portions of NCS segments; variability in most indicators was comparable in tracts containing and not containing segments.

**Conclusions:**

In a diverse urban setting, the NCS segment selection process succeeded in identifying recruitment areas that are, as a whole, representative of Queens County, for a broad range of community-level variables.

## Background

The National Children’s Study (NCS) is a prospective cohort study designed to identify preventable causes of childhood disease in the United States, with the full cohort to include 100,000 children enrolled from 105 counties (or groups of counties) across the country. A major premise of the NCS is that findings could be extrapolated to represent the American experience, and inform public policy [1-4]. Seven “pilot” or Vanguard Centers began recruitment in 2009 and Duplin County, NC and Queens County, NY were the first to enumerate and screen potential subjects residing within predetermined geographic areas, referred to as segments. These segments were selected to produce a representative subsample of the county that would, given estimated recruitment rates, result in recruitment into the study of approximately 1,000 mothers giving birth over a four year period.

The World Health Organization (WHO) has identified neighborhood contextual exposures as a central element in its Multiple Exposures Multiple Effects (MEME) framework for studying childhood health [5]. Multiple neighborhood contextual characteristics have been shown to affect a range of developmental and health outcomes across childhood and adolescence, with cognitive functioning being one of the most widely investigated. The socioeconomic composition of neighborhood residents is associated with cognitive functioning [6-11], and there is some evidence that this effect differs by race and ethnicity [8,9]. As is the case with cognitive functioning, school achievement has also been associated with neighborhood socioeconomic status (SES) [12], and gender-specific effects have been shown [13]. In addition to effects on cognitive function, a growing literature demonstrates neighborhood effects on both physical and mental health and behavior. Proximity to and quality of parks, playgrounds, and recreational facilities have been associated with physical activity, health behaviors and body size [14-24]. Similarly, indices of neighborhood walk-ability, such as population density and land use, are associated with physical activity through walking and active travel among youth [25,26]. Some studies demonstrate that physical deterioration (e.g. graffiti, litter or abandoned buildings) is associated with lower physical activity, higher rates of overweight in children, and lower parental support for children playing in local playgrounds [27-30]. Other important associations between neighborhood characteristics and physical outcomes include traffic-related respiratory symptoms [31] and injuries [32]. Among the mental health and behavioral outcomes influenced by neighborhood conditions (primarily SES-related), are psychological distress [33], substance use [34], and behavioral problems [10,35,36].

Consistent with the WHO MEME framework, one of the goals of the NCS is to understand how neighborhood environments influence child development and disease risk [1]. However, there are ethical and logistical challenges to the achievement of this goal within the context of a national, multi-site study such as the NCS, which is coordinated by a central data center. Outside of data collected nationally by the Census Bureau through the American Community Survey the availability and quality of geo-spatially aligned data describing neighborhood contexts varies tremendously across cities in the United States. In addition, our experience has been that negotiating licenses with local Governmental agencies for geo-spatial data and the sharing of geo-spatial data is often facilitated by relationship building, trust, involvement in the community and personal connections, suggesting a substantial role for local research teams in the acquisition of geo-spatial data. It is also common that licenses for such data specify that the data not be further shared with other groups, such as the NCS data center.

One approach to conducting neighborhood health studies within the NCS would be for the central NCS data repository to release to authorized investigators analytical data sets with the residential longitude and latitude of the study subjects so the investigators could create their own neighborhood context variables. This would provide the investigators with the flexibility of creating their own neighborhood definitions (e.g. to use administrative units such as postal codes or radial buffers around the address) for study subjects and of using locally available geo-spatial data to create neighborhood measures, but could compromise the confidentiality of the study subjects. Alternatively the data center could centrally perform geo-processing functions as requested by NCS investigators and provide analytical data sets that include neighborhood context variables but not residential identifiers. For analyses using unique or not universally available geo-spatial data sources, the logistics of the NCS data center sourcing and centrally negotiating data license agreements could be a serious barrier to research. Thus, strategies to protect subject confidentiality, efficiently acquire geo-spatial data and adhere to data licensing agreements will be needed to support analyses of neighborhood health effects. Other large scale national studies in the United States have taken a variety of approaches to these issues, and we suggest that a working group of interested parties be formed to study how neighborhood effect studies can best be conducted within the NCS.

From an international perspective efforts to establish procedures for the conduct of neighborhood effects research within the NCS should be cognizant of international efforts to coordinate the conduct of new large scale birth-cohort studies [37]. The World Health Organization (WHO) is currently working to strengthen, international cooperation in the conduct of birth cohort studies, with a focus on harmonizing disease outcome, biomarker and exposure measures so that study data may be pooled [37]. Under WHO’s MEME framework, the measurement of community contextual exposures is one of the “four ingredients required for the monitoring of children’s environmental health” [38]. The development of compatible methods to define and measure neighborhood contexts across birth cohort studies and cross-cultural research to identify contextual constructs that are salient across cultures and regions are areas that warrant consideration within WHO birth cohort coordination activities.

Early work from Queens has described extant data sources at the national, county and local level that can be used to estimate chemical exposures for the children enrolled in the NCS [39]. Following the WHO MEME framework, we here broaden the discussion of neighborhood environment to include the social and built environment of the Queens Vanguard site recruitment segments and Queens as a whole [1,40]. Our goal here is to identify multiple domains of neighborhood context and key variables describing the dimensions of these domains. Important considerations for conducting neighborhood health studies are whether the neighborhoods are representative of the larger area to which study results will be generalized, and whether there is sufficient variability across neighborhoods for comparisons of contextual variables to be meaningful. Consideration and analysis of these issues for neighborhood effects studies should be part of the WHO efforts to coordinate and harmonize new birth cohort studies within the MEME framework [37,38]. Here we compare the Queens segment areas to the whole of Queens to determine if the segments are representative of Queens County on the selected indicator variables and assess the extent to which the segments vary in neighborhood conditions.

## Methods

Based on the literature researchers with the Columbia University Built Environment and Health Research Group (BEH), the Columbia Children’s Center for Environmental Health and the NCS Queens Vanguard site identified nine major domains of social and built environment contexts of interest. The domains of interest and key indicator variables are described in Table 1. Available geo-spatial data from the Census and other sources have been gathered by BEH, and subsequently cleaned and geo-processed for use with the Queens Vanguard segments. Some of the data sources are available nationally, while others are unique to NYC (see Table 1).

**Table 1:** Domains of Neighborhood Characteristics, Key Variables and Data Sources

**Domain and Variables**	**Data Source**
**a. Demographic Variables**	
1). Total population	US Census 2000 SF3 file
2). Population density (10k people / km^2^)	US Census 2000 SF3 file
3). Total number of house holds	US Census 2000 SF3 file
4). Proportion below age 18	US Census 2000 SF3 file
5). Proportion above age 64	US Census 2000 SF3 file
6). Proportion Female	US Census 2000 SF3 file
7). Race	US Census 2000 SF3 file
a). Proportion White only	US Census 2000 SF3 file
b). Proportion Black only	US Census 2000 SF3 file
c). Proportion Asian-Pacific Islander only	US Census 2000 SF3 file
d). Proportion other only	US Census 2000 SF3 file
e). Proportion two or more races listed	US Census 2000 SF3 file
8). Ethnicity	US Census 2000 SF3 file
a). Proportion Hispanic	US Census 2000 SF3 file
b). Proportion foreign born	US Census 2000 SF3 file
c). Proportion linguistically isolated	US Census 2000 SF3 file
**b. Socio-economic Variables**	
1). Proportion with High school degree or beyond	US Census 2000 SF3 file
2). Proportion with a Bachelors degree or beyond	US Census 2000 SF3 file
3). Per capita income	US Census 2000 SF3 file
4). Median house hold income	US Census 2000 SF3 file
5). Proportion of people whose income is below the federal poverty level	US Census 2000 SF3 file
6). Unemployment rate	US Census 2000 SF3 file
**c. Information about Households**	
1). Average house hold size	US Census 2000 SF3 file
2). Total housing units	US Census 2000 SF3 file
3). Percent of households renting their home	US Census 2000 SF3 file
**d. Maternal Birth Statistics**	
1). Proportion of births to women <17 years old	New York City Department of Health and Mental Hygiene, Office of Vital Statistics.
2). Proportion of births to women with less than 12 years of education	New York City Department of Health and Mental Hygiene
3). Proportion of births to primiparous mothers	New York City Department of Health and Mental Hygiene
4). Proportion of mothers with late or no prenatal care	New York City Department of Health and Mental Hygiene
5). Proportion of births to mothers receiving Medicaid	New York City Department of Health and Mental Hygiene
6). Proportion of births that are low birth weight	New York City Department of Health and Mental Hygiene
7). Proportion of births that are pre-term.	New York City Department of Health and Mental Hygiene
**e. Transit Related Variables**	
1). Proportion of commuters using private cars	US Census 2000 SF3 file
2). Proportion of commuters using public transit	US Census 2000 SF3 file
3). Subway stop density (stops/km^2^)	NYC Metropolitan Transit Authority
4). Bus stop density (stops/km^2^)	NYC Metropolitan Transit Authority
5). Street network distance to central business district 1 (km)	New York State Street Centerlines
6). Street network distance to central business district 2 (km)	New York State Street Centerlines
7). Street length weighted average speed limit	New York State Street Centerlines
**f. Greenery and Playgrounds**	
1). Street trees per km^2^	New York City Department of Parks & Recreation
2). Number of playgrounds	New York City Department of Parks & Recreation
**g. Safety and Social Disorder**	
1). Pedestrians injured in car accidents	New York State Department of Transportation
2). Bicyclists injured in car accidents	New York State Department of Transportation
3). Average crime complaints per capita (2001–2004)	New York City Police Department, CompStat Crime Data.
**h. Land Use Variables**	
1). Proportion of building space that is residential	New York City Department of City Planning, PLUTO database
2). Proportion of building space that is commercial	New York City Department of City Planning, PLUTO database
3). Proportion of lot area zoned commercial	New York City Department of City Planning, PLUTO database
4). Residential-commercial land use mix	New York City Department of City Planning, PLUTO database
5). Number of buildings	New York City Department of City Planning, PLUTO database
6). Average number of floors per building	New York City Department of City Planning, PLUTO database
7). Walk-ability score	A composite score including population density, intersection density, subway and bus stop density and residential-commercial land use mix.
**i. Pollution Sources**	
1). Percentage of Tract within 805m of a TRI Site	EPA, Toxic Release Inventory Database
2). Percentage of Tract within 402m of a Stationary Point Source	EPA, National Emissions Inventory
3). Proportion of tract with 300 meters of a major trucking route	NYC Metropolitan Transit Authority
4). Proportion of tract covered by any of the above	

The overall strategy for segment selection in the NCS has been reported previously elsewhere [41]. The Queens Vanguard Center comprises 18 geographic areas, referred to as segments, which are noncontiguous, relatively homogeneous areas from which study subjects are recruited. Historical birth counts from the New York City (NYC) and New York State (NYS) Vital Statistics Registries (2000–2004) at the census tract level and NYC Housing Department data were used to predict births within census blocks. Census blocks were chosen to be representative in terms of race/ethnicity, poverty status, age distribution and foreign born status of women of child bearing age. These blocks were then combined to achieve eighteen segments that would produce 250 live births per year. The eighteen segments were selected in a two-phase stratified sampling approach that attempted to equalize the probability of selection of segments with diverse sociodemographic and other characteristics. The segment boundaries were guided by boundaries of historical neighborhoods as catalogued by the NYC Department of City Planning, and examination of proposed segment maps to ensure that selected boundaries did not cross major roadways, parks or other entities around which communities are formed. Between March-August, 2008 all dwelling units (DU) within the segments were identified (N = 11,116) and to date, 44 newly constructed DUs have been included in the sample, resulting in a total of 11,160 households [2].

Summary statistics were generated for these neighborhood context variables. Queens NCS segments were then compared to the remainder of Queens County to determine the degree to which segment selection (based on relatively few birth and demographic variables) yielded areas that were representative of Queens as a whole. Mean, median, quartile and minimum and maximum values were calculated for each variable; and segmented and non-segmented areas were compared using t-tests and non-parametric tests.

**Figure 1:**
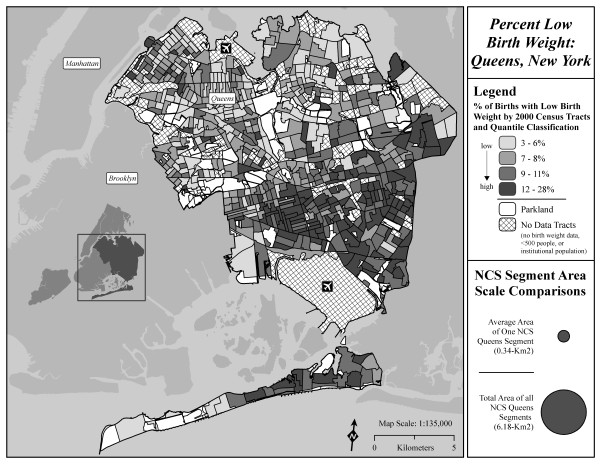
**Distribution of percent low birth weight (<2500 g) in Queens Census tracts.** In comparison to the area of Queens (281.59 km^2^) shown on the map, the circles in the legend of the maps represent the total area of the 18 segments (middle circle - 6.18 km^*2*^) and the average area of a Queens NCS segment (smallest circle – 0.34 km^2^).

**Figure 2:**
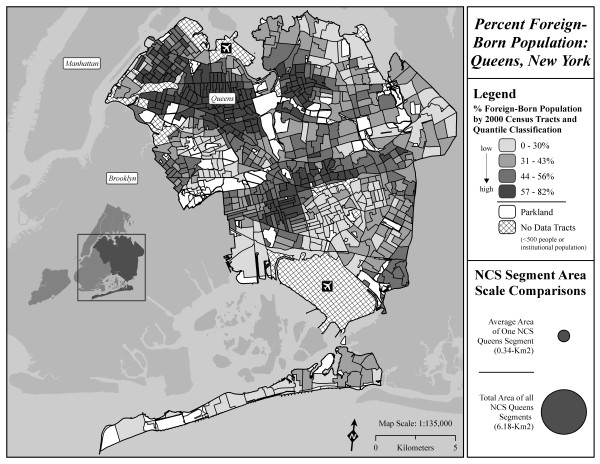
**Distribution of percent foreign-born population in Queens Census tracts.** In comparison to the area of Queens (281.59 km^2^) shown on the map, the circles in the legend of the maps represent the total area of the 18 segments (middle circle - 6.18 km^*2*^) and the average area of a Queens NCS segment (smallest circle – 0.34 km.^2^).

To preserve the confidentiality of the study subjects during the recruitment phase of the NCS, the locations of the Queens segments are not disclosed. Thus summary statistics for Census tracts that include Census blocks that are part of the segments were calculated and compared to summary statistics for Census tracts that do not include segment Census blocks (see Figures 1 and 2). To ensure the stability of summary statistics calculated at the tract level, tracts containing a total population less than 500 were excluded from analysis (n = 75); one additional tract that consisted predominantly of institutionalized individuals unlikely to include children, and this tract was also excluded from analysis. The remaining 640 tracts included in the analysis contained a total population of 2,225,761, or 99.9% of the population of Queens. A total of 43 tracts with a population of 168,503 contained NCS segments; a total of 597 tracts with a population of 2,057,258 included the remaining Queens tracts that did not contain NCS segments. Analyses in this paper did not use human subject data.

## Results

Descriptive statistics comparing Census tracts containing portions of NCS segments (N = 43) with those tracts not containing portions of the NCS segments (N = 597) are shown in Table 2. To preserve anonymity of the segments only means and inter-quartile ranges are reported in Table 2. Across the nine domains characterizing Queens communities, the NCS segment-containing tracts were, as a group quite similar to the tracts in Queens not containing portions of NCS segments. More specifically, of the 53 community indicators representing these nine domains, a statistically significant difference (p < 0.05) was found for only 7 indicators, using either a non-parametric (Mann–Whitney U) or a parametric (t-test) statistical test. The indicators with statistically significant differences were as follows: NCS segment-containing tracts had a higher proportion of the Asian and Pacific Islanders, a smaller proportion of individuals reporting that they were members of two or more races, a lower proportion of female residents, a smaller proportion of teen mothers, a smaller percentage of low-birth weight births, fewer bicyclists injured in car accidents, and a lower proportion of Tract area within a ¼ mile of a pollution point source.

**Table 2:** **Comparison of Queens Census Tracts without Segments (n=597) and With NCS Segments (n=43): Mean, Inter-Quartile Range(IQR), and IQR Ratio**^**1**^

	**Without Segments**		**With Segments**		
**a. Demographic Characteristics**	**Mean**	**IQR**	**Mean**	**IQR**	**IQR Ratio**^**2**^
1) Total population	3446	2493	3919	3313	1.33
2) Population Density	1.36	1.06	1.54	0.73	0.69
3) Number of Households	1212	870	1350	1252	1.44
4) % Population <18	24	8	24	7	0.88
5) % Population >64	13	7	12	3	0.43
6) % Female*	52	4	51	3	0.75
7) Race				3313	
a) % White only	43	48	40	32	0.67
b) % Black only	24	35	22	24	0.69
c)% Asian-Pacific Islander only**	16	21	22	25	1.19
d) % other only	11	15	11	11	0.73
e) % two or more races listed**	6	5	5	4	0.80
8) Ethnicity					
a) % Hispanic	22	23	22	17	0.74
b) % foreign born	43	25	46	28	1.12
c) % linguistically isolated	15	16	17	21	1.31
**b. Socio-economics**					
1) % with at least a High School degree	74	14	75	11	0.79
2) % with Bachelor’s degree or higher	23	15	24	12	0.8
3) Per Capita Income	19675	7885	19155	5581	0.71
4) Median Household Income	46011	16603	45261	16798	1.01
5) % Below Poverty Level	14	11	14	10	0.91
6) Unemployment Rate	8	5	7	5	1.00
**c. Households**					
1) Average Household Size	2.94	0.7	2.9	0.58	0.83
2) Total Housing Units	1265	891	1412	1290	1.45
3)% Households Renting	51	40	56	45	1.13
**d. Birth-Related:%** of Births…					
1) to Women <17*	2	2	2	2	1.00
2) to Women with < 12 Years Education.	20	14	18	12	.86
3) to Primiparous Women	35	8	36	8	1.00
4) to Women with little or no prenatal care	9	6	9	5	0.83
5) to Women on Medicaid	50	22	55	22	1.00
6) Low Birth Weight**	9	5	8	2	0.40
7) Pre-Term	13	5	12	6	1.2
**e. Transit Related**					
1) % Commuters Using Cars	47	24	45	23	0.96
2) % Commuters Using Public Transport	45	21	46	19	0.90
3) Subway Stop Density	0.49	0	0.32	0	-
4) Bus Stop Density	27	20	25	26	1.30
5) Distance to central business district 1 (km)	11	5	11	6	1.20
6) Distance to central business district 2 (km)	10	6	10	8	1.33
7) Street length weighted average speed limit	27	3	27	3	1.00
**f. Playground and Greenery**					
1) Street Trees per km^2^	1100	752	1040	491	0.65
2) Number of Playgrounds	0.36	1	0.44	1	1.00
**g. Safety and Social Disorder**					
1) Pedestrians Injured in Car Accidents	3	4	3	5	1.25
2) Bicyclists Injured in Car Accidents*^, 3^	0.71	1	1.19	2	2.00
3) Annual Crime Complaints per capita	0.016	0.004	0.016	0.005	1.25
**h. Land Use**					
1) % of Building Space – Residential	87	12	84	13	1.08
2) % of Building Space – Commercial	13	12	16	13	1.08
3) % Lot Area Zoned Commercial	12	14	11	14	1.00
4) Land Use Mix	34	35	29	30	.85
5) Number of Buildings	513	298	489	232	0.78
6) Mean Floors Per Building	2.2	0.4	2.5	0.2	0.50
7) Walk-ability	−1	4	−1	3	0.75
**i. Pollution Sources**					
1)% of Tract within 805m of a TRI Site	29	67	31	69	1.03
2) % of Tract within 402m of Point Source**	4	0	1	0	-
3) % of tract with 300m of truck route	39	40	37	51	1.28
4) % of tract covered by any of the above	54	67	53	55	0.82

*p<0.05 by Mann Whitney U **p<0.05 by t-test.

^1^ A full description of each indicator, including the data source, is provided in Table 1. Except for indicators with small values, results are rounded for display purposes to the nearest whole number; original values (with decimals) were used for purposes of statistical testing.

^2^ Inter-quartile range ratio: the ratio of the inter-quartile range (segmented tracts divided by non-segmented tracts); a value greater than 1 indicates more variability in segmented tracts than in non-segmented; a value less than 1 indicates more variability in non-segmented tracts.

Because of the relatively large number of tests involved in these comparisons, many of the seven statistically significant differences are probably not ‘significant’ in the sense that they indicate that segment tracts are not ‘representative’ of non-segment tracts. No adjustment was made for multiple comparisons; given the fact that 53 tests of each type were performed, it would be expected that approximately 3 indicators would be significantly different at the 0.05 level for each type of test—a total of seven significant differences—purely by chance.

Variability in measures among tracts is arguably as important as central tendency with respect to ‘representativeness’. If the tracts containing NCS segments were systematically less variable than the tracts not containing segments with respect to the community context indicators, the NCS segments could not be considered representative of Queens as a whole, even if the mean level of the indicators was comparable. However, an examination of the ratio of inter-quartile ranges (IQR ratio) for the two groups suggests that the two sets of tracts are in general comparable in terms of variability: as can be seen in Table 2, 25 indicators had an IQR ratio within 20% of 1 (equal variability); an additional 23 had an IQR ratio no more than 1.5 and no less than 0.5 , only 4 indicators had a ratio of <0.5 or greater than 1.5 (two indicators had inter-quartile ranges of zero, so that the IQR could not be calculated).

To provide a sense of the geographic variability of these indicators, two maps are provided, one showing the distribution of percent low birth weight (<2500 g) in Queens Census tracts (Figure 1), the other showing the distribution of percent foreign born individuals. The maps also show the approximate size of an average segment in relation to Queens as a whole, and the approximate size of all 18 segments combined.

## Discussion

Previous smaller, longitudinal birth cohorts, both in the US and internationally, have made enormous contributions to our understanding of how maternal nutrition, environmental exposures and social circumstances shape child health and development [42-47]. The NCS is designed to expand upon this prior work, at a scale that will allow for analyses of interactions between environmental pollutants, genetics, neighborhood effects and social forces [1]. This scale will facilitate the identification of determinants of childhood disease and the characterization of susceptible sub-populations of children that require a higher level of protection or interventions. Applying the WHO MEME framework to the NCS, we described several domains of neighborhood context variables that may be important determinants of child health and development. While not exhaustive, these domains represent areas of concern identified in the literature, including our own studies of neighborhood effects on health. The variables highlighted here as measures of these domains do not represent the full breadth of dimensions for these domains, but they do represent the key element, and were selected because of the availability of geo-spatial data sets with sufficient spatial resolution in the data to characterize Census tracts.

The analyses presented here document that at the tract level, the Queens NCS segments are representative of Queens overall for a large number of neighborhood level variables. The few differences identified are compatible with chance associations arising across a large number of comparisons, and there are no readily apparent processes to causally explain the differences. The block groups comprising the NCS segments were selected based on a relatively small number of socio-demographic and vital statistics. However, these variables appear to be correlated with a larger number of other socio-demographic and urban design variables, such that the tracts including portions of the NCS segments are very similar to tracts not including portions of the NCS segments. In addition, these analyses suggest that the Queens segments include a substantial amount of variation in neighborhood context variables for many domains of interest in neighborhood health studies.

Census tracts in NYC are sufficiently small that they are likely to be representative of the segments, but in recognition of disclosure risks, are sufficiently large that tabulated summary statistics won’t reveal the location of the segments. Furthermore, since women and children living in the segments are likely to experience social and environmental conditions in area adjacent to the segments, the use of Census tracts encompassing the segments partially accounts for this spatial spillover effect [48]. The analyses show that results of neighborhood context studies derived from the Queens site will likely be generalizable to Queens as a whole.

Measures of many social and economic constructs can be developed from national American Community Survey and Economic Census data and used across NCS sites, leveraging the substantial between-site variation in neighborhood contexts. In addition, other geo-spatial data-sets are often available at the municipal or county level, providing each NCS site with unique spatial data and opportunities to perform neighborhood context analyses. Geo-spatial data sets developed by NYC agencies have been extremely useful in studies of adult health in NYC [49-53] and provide unique opportunities for studying the effects of neighborhood built and social environments on child health and development [49,54].

The issue of differences in availability and quality of neighborhood contextual data across regions is amplified when one considers WHO’s efforts to harmonize and coordinate birth cohort studies internationally. Part of the efforts to standardize measures of exposure should include the identification of neighborhood/community level contextual variables that can be used across studies. This would necessitate the development of cross-cultural knowledge on how commonly used neighborhood constructs like neighborhood disorder and neighborhood-level socioeconomic conditions translate across nations. In addition, since much of the work on neighborhood contextual effects on health has been conducted in the United States and Western Europe, work on identifying additional neighborhood contextual constructs salient to other nations/regions is vital. Similarly, cross-cultural research on how the concepts of “neighborhood-level” or “community-level” are defined or are salient needs to be undertaken [40,48,55,56]. Current literature on neighborhood effects commonly uses administrative boundaries (e.g. postal codes or Census tracts) or radial or street network buffers centered on a subject’s home to define neighborhoods [48,51,55]. However, just as within a single region individual’s conceptualizations of neighborhood can vary, there are likely to be substantial differences in how individuals define neighborhoods across international contexts [57-59]. Furthermore, while the definition of a neighborhood used in research should represent the geographic scale over which a neighborhood level phenomena is thought to causally influence health, the geographic scale across which social and physical contexts affect health may vary across cultures.

## Conclusions

The WHO MEME framework identifies neighborhood and community contexts as one of the four key indicators of children’s environmental health [38]. In applying the MEME framework to the NCS, we have identified multiple domains of neighborhood context and key variables describing the dimensions of these domains that can be used in the National Children’s Study (NCS) site in Queens and many of which can be used throughout the NCS. We show that the selection of block groups to form the NCS segments in Queens using a short list of neighborhood contextual indicators (race/ethnicity, poverty status, age distribution and foreign born status of women of child bearing age) produced segments that are representative of Queens County, across many neighborhood variables. The segments also show a substantial amount of variation in neighborhood contextual variables for several domains of interest for neighborhood health studies. This suggests that unbiased studies of contextual and individual level risk factor effects on child health outcomes can be conducted within the Queens site. From a larger perspective, the NCS presents a valuable opportunity for conducting studies of the role of neighborhood context on child development and health. The development of strategies to conduct neighborhood health research and protect subject confidentiality, efficiently acquire geo-spatial data and adhere to data licensing agreements should be a priority. These issues plus the development of an understanding of the content validity of neighborhood contextual measures and the very meaning of “neighborhood” across cultures and regions should be a priority for WHO efforts to coordinate and harmonize data across birth cohorts [37].

## Abbreviations

NCS: National Children’s Study; SES: Socioeconomic status; NYC: New York City; NYS: New York State; DU: Dwelling unit; IQR: Inter-quartile range; WHO: World Health Organization.

## Competing interests

The authors are investigators and collaborators of the Queens, New York Vanguard Center of the National Children’s Study, and the Columbia Center for Children’s Environmental Health. The NCS has been funded in whole or in part with Federal funds from the National Institute of Child Health and Human Development, National Institutes of Health, under Contracts Number NICHD HHSN275200503411C/N01-HD-5-3411. The Columbia Center for Children’s Environmental Health has been supported by the National Institute of Environmental Health Sciences (5P01ES009600, 5R01ES008977, 5R01ES11158, 5R01ES012468, 5R01ES10165), and United States Environmental Protection Agency (R827027, 82860901, RD-832141). The content of this publication does not necessarily reflect the views or policies of the Department of Health and Human Services, nor does mention of trade names, commercial products, or organizations imply endorsement by the U.S. Government.

## Authors’ contributions

AR conducted the statistical analyses and wrote the paper. VAR helped interpret the data and edit the manuscript. JQ generated the geospatial measures describing Census tract characteristics. GL helped conceptualize the geospatial measures of interest and frame the Discussion section. LT provided editorial feedback on the paper and jointly directs the Queens site of the NCS. ES helped interpret the data and contributed to the writing of the manuscript. HFA managed the Queens site data, conceptualized the research project, conducted part of the statistical analyses, and helped draft the manuscript. All authors read and approved the final manuscript.

## References

[B1] Landrigan PJ Trasande L Thorpe LE Gwynn C Lioy PJ D’Alton ME Lipkind HS Swanson J Wadhwa PD Clark EB The National Children’s Study: a 21-year prospective study of 100,000 American children Pediatrics 2006 118 5 2173 2186 17079592 10.1542/peds.2006-0360

[B2] Trasande L Andrews H Goranson C Li W Barrow E Vanderbeek S McCrary B Allen S Gallagher K Rundle A Early experiences and predictors of recruitment success for the National CHildren’s Study Pediatrics 2011 127 2 261 268 21262893 10.1542/peds.2010-2334 PMC3025422

[B3] Trasande L Cronk CE Leuthner SR Hewitt JB Durkin MS McElroy JA Anderson HA Landrigan PJ The National Children’s Study and the children of Wisconsin WMJ 2006 105 2 50 54 16628976

[B4] Trasande L Landrigan PJ The National Children’s Study: a critical national investment Environ Health Perspect 2004 112 14 A789 A790 15471708 10.1289/ehp.112-1247577 PMC1247577

[B5] Briggs D Making a difference: indicators to improve children’s environmental health Indicators 2003 WHO, Geneva

[B6] Mayer S Jencks C Growing up in poor neighborhoods: how much does it matter? Science 1989 243 4897 1441 1445 17839748 10.1126/science.243.4897.1441

[B7] Breslau N Chilcoat H Susser E Matte T Liang K Peterson E Stability and change in children’s intelligence quotient scores: a comparison of two socioeconomically disparate communities Am J Epidemiol 2001 154 8 711 717 11590083 10.1093/aje/154.8.711

[B8] Brooks-Gunn J Duncan G Klebanov P Sealand N Do neighborhoods influence child and adolescent development? Am J Sociol 1993 99 353 395

[B9] Duncan G Brooks-Gunn J Klebanov P Economic deprivation and early childhood development Child Dev 1994 65 2 296 318 7516849

[B10] Chase-Lansdale P Gordon R Brooks-Gunn J Klebanov P Brooks-Gunn J, Duncan G, Alber J Neighborhood and family influences on the intellectual and behavioral competence of preschool and early school-age children Neighborhood Poverty: Vol I Context and Consequences for Children 1997 Russell Sage Foundation, New York 79 118

[B11] Chase-Lansdale P Gordon R Economic hardship and the development of five- and six-year-olds: neighborhood and regional perspectives Child Dev 1996 67 3338 3367

[B12] Leventhal T Brooks-Gunn J The neighborhoods they live in: the effects of neighborhood residence on child and adolescent outcomes Psychol Bull 2000 126 2 309 337 10748645 10.1037/0033-2909.126.2.309

[B13] Entwisle D Alexander K Olson L The gender gap in math: its possible origins in neighborhood effects Am Sociol Rev 1994 59 822 838

[B14] Farley TA Meriwether RA Baker ET Watkins LT Johnson CC Webber LS Safe play spaces to promote physical activity in inner-city children: results from a pilot study of an environmental intervention Am J Public Health 2007 97 9 1625 1631 17666701 10.2105/AJPH.2006.092692 PMC1963283

[B15] Roemmich JN Epstein LH Raja S Yin L Robinson J Winiewicz D Association of access to parks and recreational facilities with the physical activity of young children Prev Med 2006 43 6 437 441 16928396 10.1016/j.ypmed.2006.07.007

[B16] Norman GJ Nutter SK Ryan S Community design and access to recreational facilities as correlates of adolescent physical activity and body-mass index J Phys Act Health 2006 3 1 Suppl S118 S128 10.1123/jpah.3.s1.s118 28834510

[B17] Gordon-Larsen P Nelson MC Page P Popkin BM Inequality in the built environment underlies key health disparities in physical activity and obesity Pediatrics 2006 117 2 417 424 16452361 10.1542/peds.2005-0058

[B18] Pate RR Colabianchi N Porter D Almeida MJ Lobelo F Dowda M Physical activity and neighborhood resources in high school girls Am J Prev Med 2008 34 5 413 419 18407008 10.1016/j.amepre.2007.12.026 PMC2408745

[B19] Frank L Kerr J Chapman JS James Urban Form Relationships With Walk Trip Frequency and Distance Among Youth Am J Health Promot 2007 21 4 suppl 305 17465175 10.4278/0890-1171-21.4s.305

[B20] Cohen DA Ashwood JS Scott MM Overton A Evenson KR Staten LK Porter D McKenzie TL Catellier D Public parks and physical activity among adolescent girls Pediatrics 2006 118 5 e1381 e1389 17079539 10.1542/peds.2006-1226 PMC2239262

[B21] Sallis JF Nader PR Broyles SL Berry CC Correlates of physical activity at home in Mexican-American and Anglo-American preschool childre Health Psychol 1993 12 5 390 398 8223363 10.1037//0278-6133.12.5.390

[B22] Timperio A Crawford D Telford A Salmon J Perceptions about the local neighborhood and walking and cycling among children Am J Prev Med 2004 38 1 39 47 10.1016/j.ypmed.2003.09.026 14672640

[B23] Evenson KR Scott MM Cohen DA Voorhees CC Girls’ perception of neighborhood factors on physical activity, sedentary behavior, and BMI[ast] Obesity 2007 15 2 430 445 17299117 10.1038/oby.2007.502

[B24] Romero AJ Low-income neighborhood barriers and resources for adolescents’ physical activity J Adolesc Health 2005 36 253 259 15737782 10.1016/j.jadohealth.2004.02.027

[B25] Kerr J Rosenberg D Sallis JF Saelens BE Frank LD Conway TL Active commuting to school: associations with environment and parental concerns Med Sci Sports Exercise 2006 38 4 787 794 10.1249/01.mss.0000210208.63565.73 16679998

[B26] Kerr J Frank L Sallis JF Chapman J Urban form correlates of pedestrian travel in youth: differences by gender, race-ethnicity and household attributes Transp Res Part D: Transp Environ 2007 12 3 177 182

[B27] Molnar BE Gortmaker SL Bull FC Buka SL Unsafe to Play? Neighborhood disorder and lack of safety predict reduced physical activity among urban children and adolescents Am J Prev Med 2004 18 5 378 386 10.4278/0890-1171-18.5.378 15163139

[B28] Miles R Neighborhood disorder, perceived safety, and readiness to encourage use of local playgrounds Am J Prev Med 2008 34 4 275 281 18374240 10.1016/j.amepre.2008.01.007

[B29] Lumeng JC Appugliese D Cabral HJ Bradley RH Zuckerman B Neighborhood safety and overweight status in children Arch Pediatr Adolesc Med 2006 160 1 25 31 16389207 10.1001/archpedi.160.1.25

[B30] Hume C Salmon J Ball K Associations of children’s perceived neighborhood environments with walking and physical activity Am J Health Promot 2007 21 3 201 207 17233239 10.4278/0890-1171-21.3.201

[B31] Dales R Wheeler A Mahmud M Frescura A Liu L The influence of neighborhood roadways on respiratory symptoms among elementary schoolchildren J Occup Environ Med 2009 51 654 660 19448574 10.1097/JOM.0b013e3181a0363c

[B32] Macpherson A Roberts I Pless IB Children’s exposure to traffic and pedestrian injuries Am J Public Health 1998 88 1840 1843 9842384 10.2105/ajph.88.12.1840 PMC1509043

[B33] Homel R Burns A Environmental quality and the wellbeing of Children Soc Indic Res 1989 21 133 158

[B34] Ennett S Flewelling R Lindrooth R Norton E School and neighborhood characteristics associated with school rates of alcohol, cigarette, and marijuana use J Health Soc Behav 1997 38 55 71 9097508

[B35] Sampson R Groves W Community structure and crime: testing social-disorganization theory Am J Sociol 1989 94 774 780

[B36] Ingoldsby E Shaw D Neighborhood contextual factors and the onset and progression of early-starting antisocial pathways Clin Child Fam Psychol Rev 2002 5 21 55 11993544 10.1023/a:1014521724498

[B37] Children’s environmental health: coordination of new large-scale birth cohort studies [http://www.who.int/ceh/cohorts/en/]

[B38] Children's environmental health: Children's environmental health indicators [http://www.who.int/ceh/indicators/en/]

[B39] Lioy PJ Isukapalli SS Trasande L Thorpe L Dellarco M Weisel C Georgopoulos PG Yung C Brown M Landrigan PJ Using national and local extant data to characterize environmental exposures in the national children's study: Queens County, New York Environ Health Perspect 2009 117 10 1494 1504 20019897 10.1289/ehp.0900623 PMC2790501

[B40] Sampson RJ Morenoff JD Gannon-Rowley T Assessing "neighborhood effects": social processes and new directions in research Annu Rev Sociol 2002 28 443 478

[B41] Montaquila JM Brick JM Curtin LR Statistical and practical issues in the design of a national probability sample of births for the Vanguard Study of the National Children's Study Stat Med 2010 29 13 1368 1376 20527010 10.1002/sim.3891 PMC2894610

[B42] Rauh VA Whyatt RM Garfinkel R Andrews H Hoepner L Reyes A Diaz D Camann D Perera FP Developmental effects of exposure to environmental tobacco smoke and material hardship among inner-city children Neurotoxicol Teratol 2004 26 3 373 385 15113599 10.1016/j.ntt.2004.01.002 PMC3376003

[B43] Rauh VA Garfinkel R Perera FP Andrews HF Hoepner L Barr DB Whitehead R Tang D Whyatt RW Impact of prenatal chlorpyrifos exposure on neurodevelopment in the first 3 years of life among inner-city children Pediatrics 2006 118 6 e1845 e1859 17116700 10.1542/peds.2006-0338 PMC3390915

[B44] Perera FP Rauh V Whyatt RM Tsai WY Tang D Diaz D Hoepner L Barr D Tu YH Camann D Effect of prenatal exposure to airborne polycyclic aromatic hydrocarbons on neurodevelopment in the first 3 years of life among inner-city children Environ Health Perspect 2006 114 8 1287 1292 16882541 10.1289/ehp.9084 PMC1551985

[B45] Stein AD Kahn HS Rundle A Zybert PA van der Pal-de Bruin K Lumey LH Anthropometric measures in middle age after exposure to famine during gestation: evidence from the Dutch famine Am J Clin Nutr 2007 85 3 869 876 17344511 10.1093/ajcn/85.3.869

[B46] Whitaker RC Predicting preschooler obesity at birth: the role of maternal obesity in early pregnancy Pediatrics 2004 114 1 e29 e36 15231970 10.1542/peds.114.1.e29

[B47] Dubois L Girard M Early determinants of overweight at 4.5 years in a population-based longitudinal study Int J Obes (Lond) 2006 30 4 610 617 16570091 10.1038/sj.ijo.0803141

[B48] Guo J Bhat C Operationalizing the concept of neihborhood: application to residential location choice analysis J Transportation Geography 2007 15 1 31 45

[B49] Lovasi GS Quinn JW Neckerman KM Perzanowski MS Rundle A Children living in areas with more street trees have lower prevalence of asthma J Epidemiol Community Health 2008 62 7 647 649 18450765 10.1136/jech.2007.071894 PMC3415223

[B50] Purciel M Neckerman KM Lovasi G Quinn J Weiss C Bader M Ewing R Rundle A Creating and validating GIS measures of urban design for health research J Environ Psychol 2009 29 4 457 466 22956856 10.1016/j.jenvp.2009.03.004 PMC3433081

[B51] Neckerman KM Lovasi GS Davies S Purciel M Quinn J Feder E Raghunath N Wasserman B Rundle A Disparities in urban neighborhood conditions: evidence from GIS measures and field observation in New York City J Public Health Policy 2009 30 Suppl 1 S264 S285 19190579 10.1057/jphp.2008.47

[B52] Rundle A Roux AV Free LM Miller D Neckerman KM Weiss CC The urban built environment and obesity in New York City: a multilevel analysis Am J Health Promot 2007 21 4 Suppl 326 334 17465178 10.4278/0890-1171-21.4s.326

[B53] Beard JR Blaney S Cerda M Frye V Lovasi GS Ompad D Rundle A Vlahov D Neighborhood characteristics and disability in older adults J Gerontol B Psychol Sci Soc Sci 2009 64 2 252 257 19181694 10.1093/geronb/gbn018 PMC2655171

[B54] Lovasi G Quinn J Rauh V Perera FP Andrews H Garfinkel R Hoepner L Whyatt RM Rundle A Chlorpyrifos exposure and urban residential environment characteristics as determinants of early childhood neurodevelopment Am J Public Health 2010 E-published ahead of print 10.2105/AJPH.2009.168419 PMC3000714 20299657

[B55] Krieger N Chen JT Waterman PD Soobader MJ Subramanian SV Carson R Geocoding and monitoring of US socioeconomic inequalities in mortality and cancer incidence: does the choice of area-based measure and geographic level matter?: the Public Health Disparities Geocoding Project Am J Epidemiol 2002 156 5 471 482 12196317 10.1093/aje/kwf068

[B56] Lovasi G Moudon A Smith N Lumley T Larson E Sohn D Siscovick D Psaty B Evaluating options for measurement of neighborhood socioeconomic context: evidence from a myocardial infarction case–control study Health Place 2008 14 3 453 467 17950024 10.1016/j.healthplace.2007.09.004 PMC2442019

[B57] Coulton CJ Korbin J Chan T Su M Mapping residents' perceptions of neighborhood boundaries: a methodological note Am J Community Psychol 2001 29 2 371 383 11446289 10.1023/A:1010303419034

[B58] Lee B Campbell K Common ground? Urban neighborhoods as survey respondents see them Soc Sci Q 1997 78 4 922 936

[B59] Colabianchi N Dowda M Pfeiffer KA Porter DE Almeida MJ Pate RR Towards an understanding of salient neighborhood boundaries: adolescent reports of an easy walking distance and convenient driving distance Int J Behav Nutr Phys Act 2007 4 66 18088416 10.1186/1479-5868-4-66 PMC2225417

